# Novel remote electronic medication supply model for opioid-dependent outpatients with polypharmacy––first long-term case study

**DOI:** 10.1186/s12954-017-0182-x

**Published:** 2017-08-16

**Authors:** Samuel S. Allemann, Kenneth M. Dürsteler, Johannes Strasser, Marc Vogel, Marcel Stoeckle, Kurt E. Hersberger, Isabelle Arnet

**Affiliations:** 1 Pharmaceutical Care Research Group, Pharmaceutical Sciences, University of Basel, Basel, Switzerland; 2 Division of Addictive Disorders, University of Basel Psychiatric Hospital, Basel, Switzerland; 3 Department of Infectious Diseases, University Hospital of Basel, Basel, Switzerland

**Keywords:** Medication adherence, Medication management aid, Interprofessional collaboration, Substance use disorders, Old age

## Abstract

**Background:**

Patients with substance use disorders grow older thanks to effective treatments. Together with a high prevalence of comorbidities, psychological problems, and low social support, these patients are at high risk for medication non-adherence. Established treatment facilities face challenges to accommodate these complex patients within their setting. Electronic medication management aids (e-MMAs) might be appropriate to simultaneously monitor and improve adherence for these patients.

**Case presentation:**

We report the first long-term experiences with a novel remote electronic medication supply model for two opioid-dependent patients with HIV. John (beginning dementia, 52 years, 6 tablets daily at 12 am) and Mary (frequent drug holidays, 48 years, 5–6 tablets daily at 8 pm) suffered from disease progression due to non-adherence. We electronically monitored adherence and clinical outcomes during 659 (John) and 953 (Mary) days between July 2013 and April 2016. Both patients retrieved over 90% of the pouches within 75 min of the scheduled time. Technical problems occurred in 4% (John) and 7.2% (Mary) of retrievals, but on-site support was seldom required. Viral loads fell below detection limits during the entire observation period.

**Conclusions:**

Continuous medication supply and persistence with treatment of over 1.7 years, timing adherence of more than 90%, and suppressed HIV viral load are first results supporting the feasibility of the novel supply model for patients on opioid-assisted treatment and polypharmacy.

## Background

Along with the aging of the general population, the number of older users of illicit substances (or older drug users) is growing worldwide [1, 2]. Age-related comorbidities occur earlier than in the general population, and patients may be affected by chronic viral infections that can take decades to cause significant illness or death. Treatment of these conditions are expensive and require high adherence levels to be effective [3]. Medication adherence is “the process by which patients take their medications as prescribed, composed of initiation, implementation and discontinuation” [4]. Substance use itself has been reported to negatively affect adherence [5–10], and substance use disorders (SUDs) often coincide with multiple risk factors for medication non-adherence, such as psychiatric comorbidities [11], low socioeconomic status [12], lack of social support [12], unemployment [7], and unstable housing [13]. Non-adherence to medication has negative effects on health outcomes and costs [14].

Opioid-assisted treatment (OAT) is recommended for opioid dependence [15]. It is efficacious, cost-effective, and well tolerated [16]*.* In Switzerland, OAT is offered by a wide range of providers, such as general practitioners, specialized clinics, and addiction centers [17]. In the city of Basel, from the second treatment month onwards, individual take-home doses for up to 6 days per week are possible [18]. The outpatient addiction service (OAS) of the Psychiatric Hospital of the University of Basel, Switzerland, provides OAT and other medications for 220 patients, approximately 100 of which visit the service daily. The high frequency of mandatory visits and the distances between patients and providers pose a daily challenge for patients and providers alike [19].

Because existing nursing homes or home-care services are often not suited or willing to accommodate patients with SUDs, outpatient treatment and surveillance are provided as long as possible. Thanks to regular appointments, caregivers may ensure initiation and persistence with treatment, but may not be able to assure correct implementation of the dosing regimen. Many patients take their medications irregularly due to a lack of structure in their daily routine. As a result, treatment success may be compromised, resulting in health risks not only for patients but also for society. The cost of providing care to the aging older drug users is expected to increase, and innovative solutions to optimize medication management compatible with OAT are therefore required. In this context, we developed a novel medication supply model with interdisciplinary collaboration between the OAS, an HIV clinic, and the Emergency Pharmacy of Basel, in order to guarantee adherence by using an electronic medication management aid (e-MMA) for pre-packed polypharmacy located at patients’ homes. Various e-MMAs for polypharmacy exist, but only a few studies on implementing the devices in routine care have been published. Most studies are conducted outside of drug-using populations, do not describe the supply model in detail, and focus on the use and the benefits of e-MMAs for patients, such as improved safety of buprenorphine storage [20] or effects on adherence [21–25]. To our knowledge, this is the first electronic supply model of polypharmacy to older drug users worldwide. We present the first results of two cases followed over more than 2 years and draw lessons from the experiences.

## Case presentation

We present two cases of outpatients living in social housing in Basel, Switzerland, who obtained medication including opioids from the OAS Basel. After some years, conventional care and adherence to medication were questioned, especially after missed appointments and flares of HIV viral load. Both patients accepted the novel supply model with electronic monitoring of the entire medication. Both patients consented to the publication of their cases.

John was 50 years old when he entered the study on July 2, 2013. He had been diagnosed with HIV at the age of 25 as a result of intravenous drug use. A liver biopsy in 2014 showed cirrhosis and severe activity due to chronic HCV infection and alcohol abuse (at least 1 l of beer per day; METAVIR score F4, A3). He lost his girlfriend to suicide and lived with a friend who also suffered from SUD. He was unemployed and spent most of his days at home. After diagnosis of a long-QT syndrome in early 2013, he was switched from 150 mg methadone to 1200 mg (6 tablets) of long-acting morphine daily. Remaining treatment consisted of 5 tablets once daily: ritonavir 100 mg, darunavir 2 × 400 mg, tenofovir/emtricitabine 245 mg/200 mg, and pantoprazole 40 mg. Viral load reached 1000 copies per milliliter (copies/ml) during 2012 and early 2013. His viral load fell below detection limits after he was obliged to visit the OAS daily (instead of once weekly) to assure regular intake of his medication. However, he continued to miss appointments, and the situation remained unsatisfactory for him and his caregivers. Evaluation in the local memory clinic revealed a diagnosis of moderate Alzheimer’s disease accompanied a moderate depressive episode.

Mary was 46 years old when she entered the study on 18 August 2013. She had been diagnosed with HIV at the age of 19. She suffered from hypertensive cardiopathy, chronic lymphedema in both legs, and suspected chronic obstructive pulmonary disease (COPD). She had a history of hepatitis C and had been a heavy smoker for years. Her OAT consisted of liquid oral methadone (170 mg), and she received sustained-release methylphenidate (90 mg) for attention-deficit/hyperactivity disorder (ADHD). She lived with a friend. Both were not working and rarely left home. In 2012, she started to take her HIV medication only sporadically and stopped altogether in early 2013. As a result, her viral load increased sharply to over 250,000 copies/ml. A low CD4 count (< 200 × 10^9^ cells) necessitated the introduction of a prophylaxis against *Pneumocystis carinii* in summer 2013. Her caregivers convinced her to resume therapy with the same treatment as before and her viral load started to decrease. She understood the need for treatment but lacked the motivation to adhere despite intensive psychological support. At the time, additionally to methadone and methylphenidate, she was taking: lopinavir/ritonavir 200 mg/50 mg 4 tablets once daily, darunavir/emtricitabine 245 mg/200 mg 1 tablet once daily, and sulfamethoxazole/trimethoprim 800 mg/160 mg 1 tablet every Monday, Wednesday, and Friday. Her hypertension was not an issue at that time, and an approach of watchful waiting was considered appropriate with the intention not to jeopardize adherence to HIV medication.

### Novel supply model and assessments

**Fig. 1:**
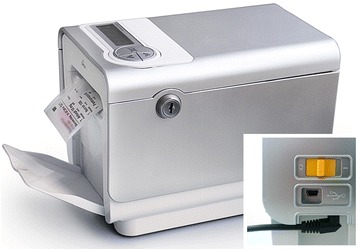
Remote-controlled, electronic medication management aid, Medido®, used in this study to dispense the unit-of-dose pouches. Notes: height × width × length, 140 mm × 140 mm × 225 mm. Weight, 1486 g. The inset shows the power cord

**Fig. 2:**
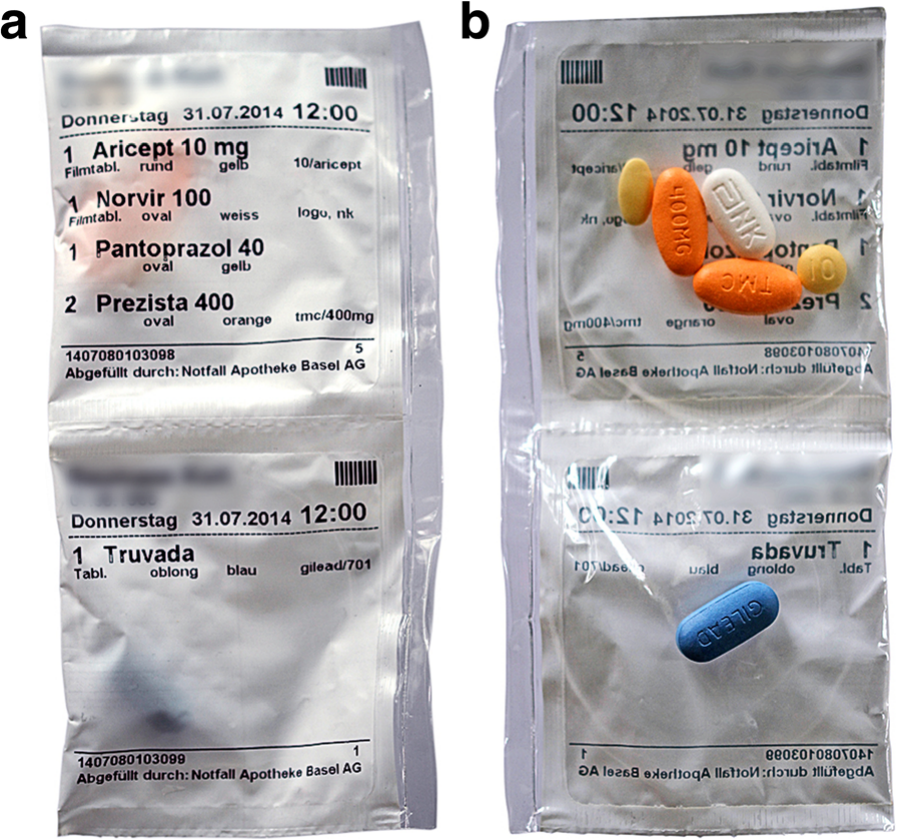
Unit-of-dose pouches with pre-packed oral solid medication from front (**a**) and back (**b**). Note: patient’s name and date of birth were concealed for privacy reasons

The core element of the novel supply model is keeping the medication for OAT in the institution and delocalizing the remaining co-medication to the patient’s home with an e-MMA (Medido®, Innospense BV, Netherlands; Fig. 1). During this pilot phase, opioids for OAT were dispensed once weekly at the OAS according to the existing law requirements. The remaining solid oral prescription medications were repackaged into unit-of-dose pouches with an Automatic Tablet Dispensing and Packaging System (ATDPS; Desk Type JV-30DE, HD-Medi, Germany). Each pouch was imprinted with the patient’s name, date of birth, and date and time for intake, as well as number, name, color, and shape of the medication contained (Fig. 2). Rolls of pouches for up to 3 weeks were placed in the e-MMA, which reminded the patients with audiovisual alerts to take their medication. A Web-based application allowed to set the time of dispense individually according to participants’ preferences. Pushing the OK button stopped the alarm and delivered the pouches with pre-packaged medication. A sensor in the dispenser registered a barcode printed on the top of each pouch and cut the pouches accordingly. Date and time of delivery were simultaneously recorded with mobile technology. Delivery of doses ahead of schedule (so-called pocket-doses) was feasible by pushing the OK button for 5 s. This feature enabled patient mobility, i.e., to be outside of home during scheduled intake times.

**Fig. 3:**
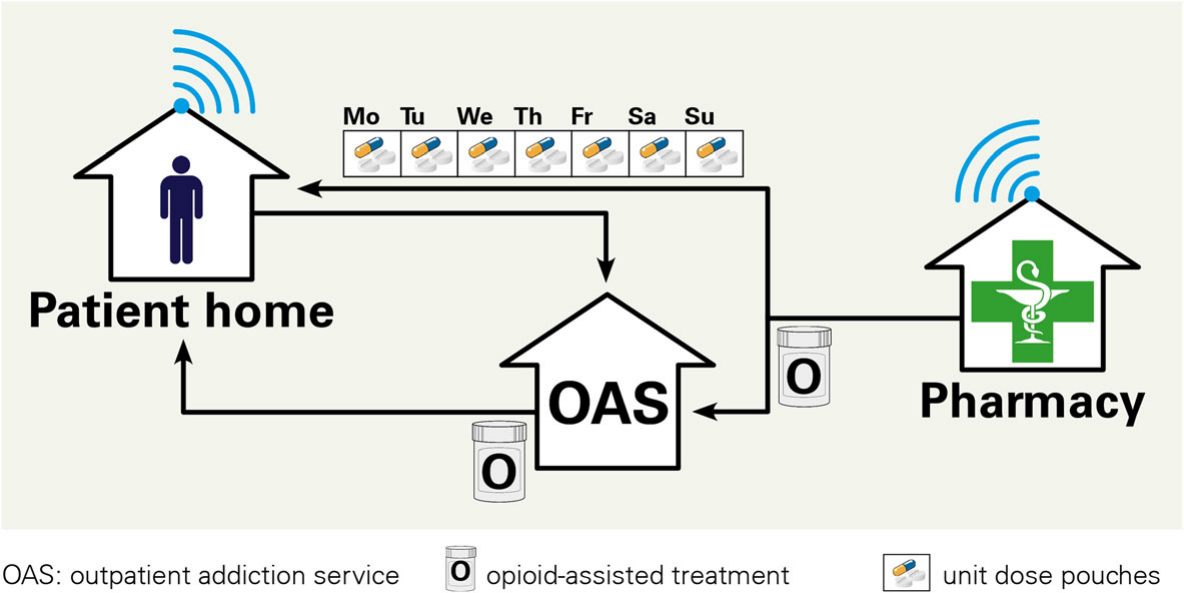
Novel medication supply model where the community pharmacy provides unit-of-use pouches (medication roll Mo-Su) with all solid oral medications directly to patient home, except opioids for opioid-assisted treatment (OAT) and methylphenidate (S). The pouches are loaded into a lockable, remote-controlled dispenser that can be programmed according to a patient’s medication schedule. The dispenser records dates and times of medication retrievals and wirelessly transmits them to a server (blue waves). Patients obtain the opioid agonist therapy (S) from the outpatient addiction service (OAS) in regular intervals, at least once weekly, according to local law requirements

The e-MMA was installed at the patients’ homes by the responsible caregiver of the OAS and a pharmacist from the Pharmaceutical Care Research Group (Fig. 3). Patients were instructed in detail about its proper use. They were also given a written manual including a telephone hotline number in case of problems with the dispenser. The hotline was operated by a pharmacist of the research group (SA or IA) during weekdays and by the Emergency Pharmacy during weekends and public holidays. Every 3 weeks, medications were repackaged according to the current treatment plan and the e-MMA was refilled during a pre-scheduled visit at the patient’s home. If a patient failed to retrieve a dose from the dispenser within 75 min after the predefined time of intake, or in case of malfunctioning, the dispenser automatically sent an alert SMS to the hotline number. The pharmacist then contacted the patient by phone, inquired the situation, acted accordingly (either by remote action or by visiting the patient at home), and made sure that medication intake had been warranted. Primary outcomes were taking adherence assessed by electronic monitoring and HIV status (viral load, CD4 count) assessed during routine visits in the HIV clinic. Electronic adherence data was analyzed and graphed with the statistical software R [26]. For taking adherence, we calculated frequencies of pre-dispense (doses dispensed before the scheduled time), regular dispense (doses dispensed during the 75-min scheduled interval), late dispense (doses dispensed more than 75 min after the first alarm), forgotten doses (dispensed remotely after pharmacist intervention), and erroneous dispense (errors during dispense due to technical problems).

### Follow-up and outcomes

The e-MMAs were installed in the kitchen (John) and in the living room (Mary). Dispense times were scheduled in line with consistent habits of daily life, i.e., 12 pm for John (first meal of the day) and 8 pm for Mary (watching TV). John was followed for 659 days. At the time of drafting of this article, Mary is still using the e-MMA. We present data from 953 days. Adherence was electronically monitored during 655 days (99.2%, John) and 911 days (95.6%, Mary; Table 1). Missing days (John, 0.8%; Mary, 4.4%) were due to technical problems with the dispenser.

**Table 1:** Description of the electronic adherence monitoring for John and Mary

	John	Mary
Days of follow-up	659	953
Number of roll replacement during refill visits	31	46
Days with electronic monitoring	655 (99.2%)	911 (95.6%)
Regular dispense	615 (94.0%)	843 (92.2%)
Pre-dispense	1 (0.2%)	5 (0.5%)
Late dispense	8 (1.2%)	0
Forgotten	4 (0.6%)	0
Dispensed with errors	26 (4.0%)	66 (7.2%)
Resolved remotely	25	60
Resolved at patient’s home	1	6

*Regular dispense* doses dispensed during the 75-min scheduled interval, *pre-dispense* doses dispensed before the scheduled time, *late dispense* doses dispensed more than 75 min after the first alarm, *forgotten* doses dispensed remotely after pharmacist intervention, *erroneous dispense* technical errors during dispense

**Fig. 4:**
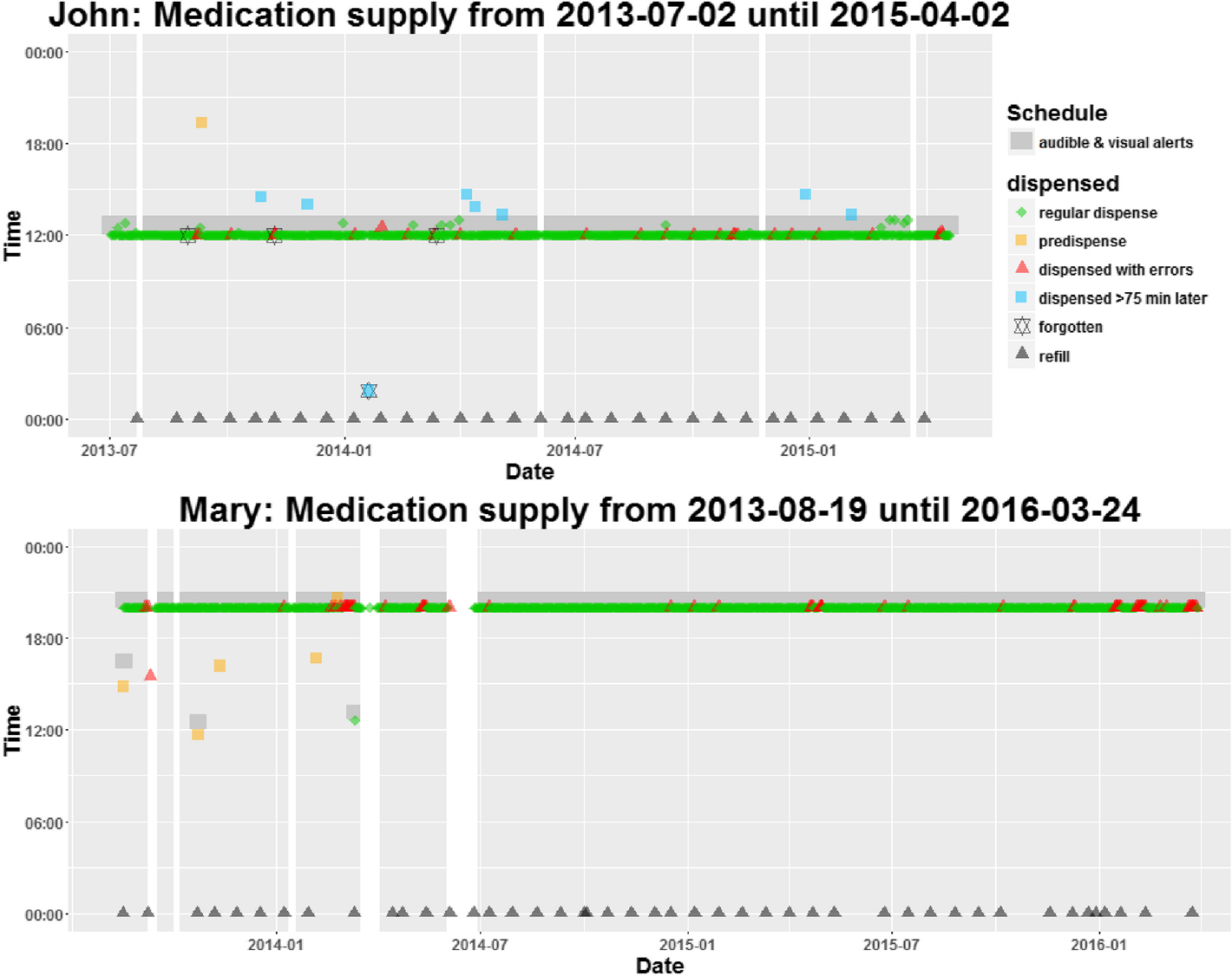
Time of medication dispense for John (659 days) and Mary (953 days) recorded with electronic monitoring. White areas are days with missing electronic monitoring (John, 0.8%; Mary, 4.4% of all days)

**Fig. 5:**
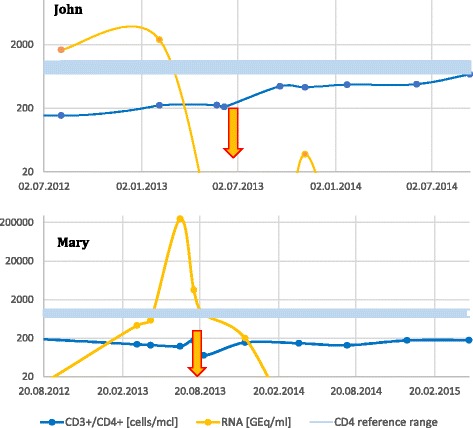
HIV viral load and CD4 cell count of John and Mary. Start of electronic monitoring is marked with an arrow

Pill burden of John was reduced by 1 tablet through substitution of darunavir 2 × 400 mg with 1 × 800 mg. For dementia, a therapy with the acetylcholine-esterase inhibitor donepezil was initiated in October 2013 and subsequently increased to the maximal dose of 10 mg. John was satisfied with the treatment and reported no adverse events. Still, he expressed concerns regarding his persistent neurocognitive problems––disorientation and forgetfulness. He retrieved 8 doses (1.2%) more than 75 min after the scheduled time and forgot to retrieve 4 doses (0.6%, Table 1 and Fig. 4). This deviation was due to an appointment or a visit at the OAS, preventing him from being back home in time for the scheduled intake. His flat mate would sometimes retrieve the pouches and leave them on the counter for him (frequency not known). Errors during dispense of the pouches typically coincided with the end of a medication roll and did not require any intervention. Dementia remained stable (assessment in spring 2014), and the pattern of retrieved pouches from the dispenser did not change. With the exception of a blip in November 2013, his viral load remained suppressed below 20 copies/ml and CD4 cells continued to rise (Fig. 5). In April 2015, his flat mate suddenly died and the patient decided together with the care staff of the OAS that he would not continue to live independently. He moved to a supervised care home that adopted his medical care, and medication supply with the dispenser was therefore terminated.

During the first months, Mary experienced several technical problems. Pouches sometimes got stuck in the dispenser or the dispenser cut the pouches in the wrong area, which required a visit to the patient for reconfiguration of the dispenser. Despite these issues, the patient was grateful for the intervention and reported regular intake of the medication. In early 2014, viral load fell below detection limits and CD4 counts started to rise (Fig. 5). After 1 year, the dispenser was replaced and technical difficulties that required the attention of the pharmacist disappeared almost completely (Fig. 4). In instances where technical problems still caused the device to cut pouches improperly, the patient helped herself using scissors. Although she was requested to immediately call the hotline in case of technical issues, Mary only mentioned them during the refill visits every 3 weeks.

In December 2015, Mary mentioned that when she was busy in the flat and the alarm would ring, she would sometimes press the OK button to retrieve the pouches but would walk away and go back to her business. Sometimes, she would then forget to take the medications later and save the pouches in a drawer. Between May and December 2015, she reportedly skipped medication intake on 21 days (10% over 8 months). Because viral load was constantly suppressed and CD4 counts recovered, prophylaxis of *P. carinii* was discontinued in September 2015. In spring 2016, home visits for dispenser refill were discontinued. The medication rolls were delivered to the OAS where Mary would pick them up during her regular visits and load them into the dispenser at home. At the time of publication of this article (August 2017), appointments were kept and the laboratory results were satisfactory.

## Conclusions

A novel medication supply model with electronic adherence monitoring of polypharmacy showed sustained treatment implementation and suppressed viral loads in two opioid-dependent HIV patients over 1.7 and 2.5 years, respectively. The drop of viral loads started when patients resumed therapy after intervention of their caregivers. It has been demonstrated that adherence interventions for long-term treatments need regular follow-ups to remain effective [27]. However, the intensive care to assure adherence for John was not sustainable on the long term, and correct implementation of the treatment regimen was not guaranteed for Mary. Our novel supply model offered a sustainable solution to assure adequate implementation and persistence with treatment. In line with our findings, larger trials of such devices suggest an improvement of adherence and clinical outcomes for patients with kidney transplants [25] and schizophrenia [23]. These trials, however, were usually of short duration and did not focus on the implementation of dosing regimens. Additionally, repackaging of medications in unit-of-use pouches might prevent disturbance in case of changes in treatment, such as the up-titration of anti-dementia therapy or initiating of preventive and irregular treatment, e.g., the prophylaxis of *P. carinii*. Adapting the content of the pouches is possible without modifying the intake habits. This insures that changes in treatment do not coincide with variable intake times and thus might prevent non-adherence.

Electronic monitoring makes changes in dosing patterns instantly apparent and allows for a timely intervention. Feedback from electronic monitoring has been shown to effectively improve adherence [28]. The only prerequisite is a system entirely reliable and without deficiencies to avoid interference with measurements. We experienced technical problems that compromised monitoring and increased workload for the care staff. These were unpredictable, not reproducible, and complicated the care process. As a consequence, caregivers received unsuspected alerts that could not be ignored. Nevertheless, patients declared satisfaction with the novel supply model, probably because the technical problems did not jeopardize medication intake.

Our study has several strengths. First, we included patients from a population with a high probability of non-adherence and a high prevalence of time-sensitive medication regimens, such as highly active anti-retroviral therapy (HAART) for HIV. Thus, the success of our intervention in these complex patients demonstrates the potential of our supply model. Second, we measured adherence to polypharmacy. Typically, devices for electronic monitoring are designed for single preparations. The monitoring of polypharmacy thus requires multiple devices and may complicate the management of medications. With our e-MMA, all medications were dispensed in unit-of-use pouches, which enhanced the likelihood of concurrent intake.

We acknowledge some limitations. First, measuring adherence with the e-MMA might overestimate adherence because medication retrieval does not equal ingestion. Literature suggests that electronic monitoring might underestimate adherence [29], although the contrary has also been argued. The latter seems more plausible in our cases. Mary, for example, retrieved all of her pouches on time but set them aside and forgot to take at least 10% of them during an 8-month period. The greater the distance (time and place) between electronic monitoring and actual ingestion of the medication, the higher the risk of false-positive results. With the electronic dispenser, the signal is generated when patients press the button to stop the alarm and to retrieve the pouches. During the few seconds of dispensing and cutting the pouch, the patient may walk away and forget the intake later on. Furthermore, patients or other persons living in the same household might press the button to stop the alarm without the intention of taking their medication. Consequently, intentional non-adherence must be ruled out before using this kind of an e-MMA. Other systems, such as electronic punch cards (POEMS [30]), measure the emptying of a cavity directly before ingestion and are thus less likely to overestimate actual intake of tablets and distort the measurement of adherence.

Second, although John and Mary experienced a benefit from the dispenser, case reports cannot generate results to claim effectiveness of an intervention. Additionally, the generalizability of our results is limited. We evaluated the e-MMA in two patients that matched the envisaged target groups suggested in a qualitative study of the dispenser [31]. Living conditions like mobility could pose a barrier to acceptance of the stationary dispenser [31]. This might be less of an issue in opioid-dependent patients who often have no employment. Recent data from a Swiss survey indicate unemployment rates of 50% among patients with OAT [19]. However, patients not matching the envisaged target groups may also benefit from the novel supply model and further studies should evaluate this.

Finally, we did not evaluate the financial implications of the novel supply model. Costs for repackaging of medications are reimbursed in Switzerland for patients with polypharmacy (i.e., more than 3 medications during 4 months). Costs of the e-MMA and additional costs for service and support are currently not reimbursed by health insurances. However, savings from improved adherence might offset the additional costs, as shown in previous studies where better adherence resulted in significant cost savings [32, 33].

Continuous medication supply and persistence with treatment over more than 1.7 years, timing adherence of more than 90%, and suppressed HIV viral load are the first results supporting the feasibility of the novel supply model. Further trials should aim at evaluating the effectiveness of the supply model in terms of clinical, humanistic, and economic outcomes and in patients that do not necessarily match the envisaged target groups.

## Abbreviations

(e-)MMA: (Electronic) medication management aid
ATDPS: Automatic Tablet Dispensing and Packaging System
HAART: Highly active anti-retroviral therapy
HIV: Human immunodeficiency virus
OAS: Outpatient addiction service
OAT: Opioid-assisted treatment
